# Indications for and complications of intestinal stomas in the children and adults at a tertiary care hospital in a resource-limited setting: a Tanzanian experience

**DOI:** 10.1186/s12876-019-1070-5

**Published:** 2019-08-28

**Authors:** Alicia Massenga, Alfred Chibwae, Aloyce A. Nuri, Merchades Bugimbi, Yasin K. Munisi, Ramadhani Mfinanga, Phillipo L. Chalya

**Affiliations:** 1 Department of Surgery, Bugando Medical Centre, Mwanza, Tanzania; 2 Unit of Pediatric Surgery, Bugando Medical Centre, Mwanza, Tanzania

**Keywords:** Intestinal stomas, Indications, Complications, Management, Tanzania

## Abstract

**Background:**

An intestinal stoma, though a life-saving procedure on the care of many gastrointestinal conditions, carries significant number of complications. This study describes the common indications, complications, and management of stomas and identifies the factors that are associated with these complications in a tertiary care hospital in Tanzania.

**Methods:**

A cross-sectional study of patients with intestinal stomas was conducted at Bugando Medical Centre (BMC) between July 2016 and June 2017. Ethical approval to conduct the study was obtained from relevant authority before the commencement of the study.

**Results:**

A total of 167 patients (M: F = 1.2:1) were enrolled in the study. The mean age at diagnosis was 0.6 ± 1.4 years for children and mean age for adults was 36.7 ± 15.8 years. Anorectal malformation (110, 89.4%) was the most common indication for intestinal stoma formation in children, while bowel perforation (14, 31.8%) was the main indications in adults. The sigmoid colon (137, 82.0%) was the most common anatomical site for stoma formation followed by the ileum (18, 10.8%). Stoma prolapse (18, 41.9%) was the most frequent complication of a stoma, whereas, surgical site infection (9, 34.6%) was the most frequent complication after stoma closure. Thirty five (26.7.%) of the children developed stomal complications, while only 8 (22.2%) of the adults developed complications. The level of training of operating surgeon and timing of surgery were the main predictors of stoma-related complications (*p* < 0.034 and 0.013), whereas the level of training of the operating surgeon and the type of stoma closure were significantly associated with the complications related to stoma closure (*p* < 0.001).

**Conclusion:**

The intestinal stomas performed at BMC are associated with various complications, which in turn, become a burden to the patients. The insights observed in the current study may apply to other tertiary hospitals in Tanzania and Africa at large. We suggest that the keystones for improvement and control in the formation and complications of intestinal stomas are the following; colostomy formation should rarely be done in transverse colon, the procedure should be carried out by senior doctors (specialist) or junior doctors under close and direct supervision of the specialists, using proper meticulous techniques, and the need to determine and/or improve techniques for early detection of complications.

## Background

An intestinal stoma has long been one of the most commonly performed life-saving surgical procedure worldwide and plays an important role in the management of congenital and acquired gastrointestinal conditions [1]. The major reasons for performing stoma are to divert stool flow, protecting anastomotic site, bowel decompression, or a combination of these indications [2, 3]. The commonly performed ostomies in surgical practice include colostomy and ileostomy, however, there are many other variations such as the rare jejunostomy that can be created to decompress, lavage, and divert gut contents [4, 5].

Indications for intestinal stoma in children differ from those in older persons. Unlike adults, stoma formation in children, most of the time is done as a temporary surgery, as an option of management of congenital malformation of the intestines [5]. Hirschsprung’s disease and anorectal malformation are among the main diagnoses for stoma formation in kids [6]. In adults, other conditions such as volvulus, diverticulitis, trauma, and malignancies occasionally, require stoma formation as part of their management [3, 6].

In spite of the fact that intestinal stoma creation is a procedure which saves live in the care of many gastrointestinal conditions, it’s attendant morbidity and mortality have been the subject of many studies [3, 5]. When incorrectly constructed, a stoma can complicate or delay the definitive management of these malformations and can even lead to death [7]. A variety of complications associated with stoma formation and its closure have been reported [3, 7]. Complications following the creation of an intestinal stoma are experienced by 20–70% of the patients and are divided into early complications (up to 30 days after operation) such as ischemia, hemorrhage and infection, and late complications (more than 30 days after operation) such as stenosis, fistula formation, prolapse, hernia formation, colonic and small bowel obstruction and denuded peristomal skin [4, 7, 8]. Early complications are frequently technical in nature and may require immediate intervention, while late complications may be the result of early complications but more often are part of the natural history of a stoma [5].

Factors affecting the type and frequency of complications include surgical specialty, surgeon experience, emergency versus elective stoma creation, appropriate preoperative marking and patient education, patient issues such as age, obesity, diabetes, and ability to care for stoma [3].

Unlike in so many parts of Africa which rely on intestinal stoma to treat a variety of gastrointestinal and abdominal conditions, in developed countries, primary definitive surgery is largely performed instead to avoid the complications associated with stoma formation and its closure. The intestinal stoma has continued to be a life-saving procedure in the management of these conditions [7].

Despite of this common procedure being performed in the care of many gastrointestinal conditions, there is a paucity of information regarding the complications that have been observed following the intestinal stoma procedure at our local setting. Therefore, the current study was conducted to describe the common indications, complications and their management and to identify the factors that are associated with the post-procedure complications at BMC, a tertiary care hospital in Tanzania. Results from this study give insight on the complications and measures that should be taken to alleviate the conditions in tertiary health centers.

## Methods

### Study design and setting

This was a descriptive cross-sectional study that was conducted in the surgical department at BMC between July 2016 and June 2017. BMC is a tertiary care and teaching hospital for the Catholic University of Health and Allied Sciences-Bugando (CUHAS-Bugando) and has 950 beds. It is located in Mwanza city along the shore of Lake Victoria in the northwestern part of Tanzania. BMC is one of the four largest tertiary care hospitals in the country and serves as a referral center for tertiary specialist care for a catchment population of approximately 14 million people from neighboring regions in the northwestern part of Tanzania.

### Study population

The study population included all patients who were admitted to BMC who required intestinal stoma formation for a variety of gastrointestinal and abdominal conditions. All patients were admitted through emergency medicine and surgical outpatients departments (SOPD) and underwent surgery for various reasons. Patients operated earlier before undertaking this study or who were operated in other hospitals and referred to BMC for either definitive surgery or with complications of stomas, were excluded from the study. All patients who met the inclusion criteria were enrolled in the study. In each case, a detailed history and physical examination were carried out followed by relevant investigations. Resuscitation of the patients who were admitted on emergency basis was done with intravenous fluids, nasogastric suction, and intravenous antibiotics; the stoma was constructed after confirming the diagnosis. Patients with congenital anomalies were subjected to initial stoma that were done as bypasses or for decompression of the bowel followed by definitive (curative) surgery and eventual stomal closure, whereas patients with acquired intestinal conditions, had their stoma done during a colectomy or other intra-abdominal procedure that was part of a definitive operation. Different types of stomas performed included the loop, double-barrel, and end stoma made on either the colon (transverse or sigmoid) or small bowel (ileum or jejunum). Transverse colostomies were performed using a right transverse incision taken through the lateral part of the rectus-abdominis muscle. Sigmoid colostomies were performed using a left iliac incision. In the loop intestinal stoma, the stoma was brought out as a loop, and the loop was prevented from retraction by using a rubber tube passed under the mesenteric border to be removed on the second or third post-operative day. In the double-barrel type of colostomy, the bowel loop was completely transected and the two separate ends (i.e. the proximal and distal ends) were brought on the surface through the same opening and anchored to the parietal wall using 2–0 or 3–0 polyglactin sutures. Stomas were done by the operating surgeon in all patients. The operations were performed either by senior doctors (specialists) or junior doctors (surgical registrar s/residents) under either direct or indirect supervision of the senior doctors. An appliance (stoma bag) was applied immediately after operation. Stoma care was explained to patients/parents by the concerned doctor and staff nurse during the stay of the patient. Our facility has also an enterostomal nurse who provides routine education regarding stoma care. Patients were followed up until discharge or death and at three month interval until stoma closure after definitive (curative) treatment. During this period, patients were screened for presence or absence of complications. Stomal complications were assessed in both inpatients in the ward and outpatients at SOPD. After definitive treatments, all patients, depending on the purpose of the stoma formation, were planned for stomal closure. All patients who underwent stomal closure underwent bowel preparation preoperatively: Mechanical bowel preparation after low-residue diet for 2–3 days. Addition to that, they received intravenous antibiotics preoperatively. In many of loop colostomies, the bowel was transected before doing anastomosis, while in some, a wedge resection of the loop was done. Seromuscular two layers technique with 2–0/3–0 polyglactin was done for establishing bowel continuity. The abdomen was closed in double layers and in the same setting skin was closed. All stomal closures were done without drains. After stomal closure, followed up of all patients was done after stoma closure, up to death or 3-months in case of survivors. During this time the presence of complications was recorded. Coded data collection sheets were used to collect required information. Data entered in questionnaires were demographic details, indications, the purpose of the stoma formation (i.e. temporary or permanent), types of stoma, anatomical location of the stoma, level of training of the operating surgeon, time interval between stoma placement and its reversal, complications of stoma formation and closure.

### Analysis of statistical data

Statistical Package for Social Sciences (SPSS) version 20.0 was used for analysis of the data. The significance of association between the dependent (outcome) and independent (predictor) variables was tested by using Chi-square (χ2) test. Statistical significance was considered as *p* < 0.05. Variables which were subjected to multivariate logistic regression analysis are the once that were statistically significant in univariate analysis. Analysis of multivariate logistic regression was used to determine independent variables which predicted the complications occurred after surgery.

## Results

### Demographic characteristics

During the period of study, a total of 172 patients with conditions who required stoma formation were diagnosed and treated at BMC. Of these, five patients were excluded from the study due to failure to meet the inclusion criteria. Thus, 167 patients were available for the final analysis (Table 1). The age at diagnosis ranged from 1 day to 76 years. The majority of patients, 131 (78.4%) were children, less than 18 years old with mean + \-standard deviation age of 0.6 ± 1.4 years. Mean + \-standard deviation age for adults was 36.7 ± 15.8 years. Ninety (53.9%) of them were males, and 77 (46.1%) were females giving a male to female ratio of 1.2:1.

**Table 1:** Distribution of patients according to indications for intestinal stomas formation

Indications	Categories	Number of patients	Percentage
Congenital diseases (N1 = 123, 73.7%)	Anorectal Malformations	110	89.4
	Hirschsprung’s disease	12	9.8
	Colonic atresia	1	0.8
	Total	123	100
Acquired diseases (N2 = 44, 26.3%)	Bowel perforation	14	31.8
	Mechanical bowel obstruction	10	22.7
	Anal/penetrating abdominal injuries	7	15.9
	Neoplastic causes	5	11.4
	Anastomotic leak	5	11.4
	Post-haemorrhoidectomy faecal incontinence	2	4.5
	High fistula in ano	1	2.3
N1 + N2 = 167	Total	44	100

### Indications for intestinal stoma formation

Congenital anomalies were the main indication for intestinal stomas and accounted for 123 (73.7%) patients, while 44 (26.3%) patients had acquired diseases. However, anorectal malformation (89.4%) was the most common indication for intestinal stomas in children, followed by Hirschsprung’s disease (9.8%), while bowel perforation (31.8%) followed by mechanical bowel obstruction (22.7%) were the main indications for intestinal stomas in adults (Table 1). Patients who had congenital anomalies were younger, and they presented between 1 day and 6 months (mean 3.7 ± 1.4 months) compared with those children who had acquired diseases presented between 1 month and 12 years of age (mean 5.8 ± 2.6 months).

### Operative characteristics for intestinal stoma formation

Among the anatomical sites and types of stoma, sigmoid double barrel colostomy was the most commonly performed in 91 (93.8%) children and in 11 (61.1%) adults (Table 2). The majority of stomas, 157 (94.0%) were constructed for temporary purposes. Only ten (6.0%) patients had permanent stomas because of anorectal cancer and gangrenous bowel due to inferior mesenteric ischemia in three patients each respectively. Long-segment Hirschsprung’s disease in two patients and the remaining patients had permanent stomas because of incontinence due to severe spinal bifida and severe spinal cord injury in one patient each respectively. Most of the children, 73 (55.7%) patients, were operated on an emergency basis, while 26 (72.2%) adults had emergency colostomy done. The sigmoid colon was the most common anatomical site for stoma formation in 118 (90.1%) children and in 19 (52.8%) adults, while the second common anatomical site for stoma formation in children was transverse colon 6 (4.6%), and in adults was ileum 13 (36.1%). The double-barreled colostomy was the commonest type of stoma performed in 97 (74.0%) children and 18 (50.0%) adult patients. Most of the surgeons performing these colostomies were juniors 133 (79.6%), while seniors performed 34 (20.4%) of the stomas (Table 3).

**Table 2:** Anatomical sites distribution versus to the types of stoma

	Types of stoma							
Anatomical sites of stoma	Children < 18 years				Adults ≥ 18			
	Loop N (%)	Double barrel N (%)	End N (%)	Spectacle N (%)	Loop N (%)	Double barrel N (%)	End N (%)	Total N (%)
Jejunum	1 (7)	1 (1)	0 (0)	0 (0)	0 (0)	0 (0)	1 (6)	3 (2)
Ileum	1 (7)	1 (1)	3 (21)	0 (0)	0 (0)	6 (33)	7 (44)	18 (11)
Transverse colon	1 (7)	4 (4)	1 (1)	0 (0)	0 (0)	1 (6)	2 (13)	9 (5)
Sigmoid colon	12 (80)	91 (94)	10 (71)	5 (100)	2 (100)	11 (61)	6 (38)	137 (82)
Total	15 (9)	97 (58)	14 (8)	5 (3)	2 (1)	18 (11)	16 (10)	167 (100)

**Table 3:** Distribution of patients according to operative characteristics

Operative characteristics	Children less than 18 years	Adults more than 18 years	Total
	N (%)	N (%)	
Indications			
• Congenital conditions	123 (94)	0 (0)	123 (74)
• Acquired conditions	8 (6)	36 (100)	44 (26)
Purpose of stoma			
• Temporary	130 (99)	27 (75)	157 (94)
• Permanent	1 (1)	9 (25)	10 (6)
Timing of surgery			
• Elective	58 (44)	10 (28)	68 (41)
• Emergency	73 (56)	26 (72)	99 (59)
Rank of the surgeon			
• Junior	105 (80)	28 (78)	133 (80)
• Senior	26 (20)	8 (22)	34 (20)
Anatomical site of the stoma			
• Jejunum	2 (2)	1 (3)	3 (2)
• Ileum	5 (4)	13 (36)	18 (11)
• Transverse colon	6 (5)	3 (8)	9 (5)
• Sigmoid colon	118 (90)	19 (53)	137 (82)
Type of stoma			
• Loop	15 (12)	2 (6)	17 (10)
• End ± Hartman procedure	14 (11)	16 (44)	30 (18)
• Double barreled	97 (74)	18 (50)	115 (69)
• Spectacles	5 (4)	0 (0)	5 (3)

### Complications related to stoma formation

Forty three (25.7%) of the patients developed complications following colostomy formation. Thirty five (81.4%) of the children developed stomal complications, while only 8 (18.6%) adults developed complications. Generally, 33 (76.7%) complications occurred in sigmoid colon, 6 (13.9%) occurred in ileum, and the remaining 3 (6.9%) and 1 (2.3%) complications occurred in transverse colon and jejunum respectively. In children, most of complications 29 (82.8%) occurred in sigmoid colon. In adults complications occurred in ileum and sigmoid colon, each had 50% complications (Table 4). In the 5 patients who had a loop colostomy there were 29% complications, in the 8 patients with an end colostomy there were 26.7% complications, in the 29 patients with a double barrel colostomy there were 25.2%, and in the 1 patient with a spectacle colostomy there were 10% complications.

**Table 4:** Distribution of stoma formation related complications versus stomal sites (*N*= 43)

Stoma formation related complications	Anatomical site of stoma						Total N (%)
	Children				Adults		
	Jejunum (%)	Ileum (%)	Transverse colon (%)	Sigmoid colon (%)	Ileum (%)	Sigmoid colon (%)	
Denuded peristomal skin	0 (0)	0 (0)	0 (0)	2 (7)	0 (0)	0 (0)	2 (5)
Stoma Prolapse	0 (0)	0 (0)	2 (67)	16 (55)	0 (0)	0 (0)	18 (42)
Surgical site infection	0 (0)	1 (50)	0 (0)	3 (10)	2 (50)	1 (25)	7 (16)
Bleeding	0 (0)	0 (0)	0 (0)	1 (3)	0 (0)	0 (0)	1 (2)
Retraction	0 (0)	0 (0)	1 (33)	1 (3)	1 (25)	0 (0)	3 (7)
Stenosis	1 (100)	1 (50)	0 (0)	3 (10)	0 (0)	0 (0)	5 (12)
Obstruction	0 (0)	0 (0)	0 (0)	2 (7)	0 (0)	0 (0)	2 (5)
Necrosis	0 (0)	0 (0)	0 (0)	0 (0)	0 (0)	1 (25)	1 (2)
Peritonitis	0 (0)	0 (0)	0 (0)	0 (0)	1 (25)	2 (50)	3 (16)
Parastomal hernia	0 (0)	0 (0)	0 (0)	1 (3)	0 (0)	0 (0)	1 (2)
Total (N)	1 (3)	2 (6)	3 (9)	29 (81)	4 (50)	4 (50)	43 (100)

The overall complications rate was significantly higher in stomas performed on emergency basis than that performed electively (*p* = 0.013). The complication rate was also found to be significantly higher (30%) in stomas performed by junior doctors than in those performed by senior doctors, (8.8%), (*p* = 0.034). Also, the overall complications rate in the current study was found to be statistically significantly higher (50%) in transverse colostomies than in sigmoid colostomies (22%), (*p* < 0.001) (Table 5).

**Table 5:** Predictors of stoma-related complications according to univariate and multivariate logistic regression analysis

Independent variable	Complications		Univariate analysis			Multivariate analysis		
	Present	Absent	OR	95% CI	*p*-value	OR	95% CI	*p*-value
Age (years)								
• < 18	35 (81)	96 (77)	1					
• ≥18	8 (19)	28 (23)	1.34	0.54–3.77	0.675			
Sex								
• Male	20 (22)	70 (78)	1					
• Female	23 (30)	54 (70)	0.67	0.33–1.35	0.260			
Pre-morbid illness								
• Present	4 (29)	10 (71)	1					
• Absent	39 (26)	114 (75)	1.12	0.65–6.98	0.764			
Indications								
• Congenital	32 (26)	91 (74)	1					
• Acquired	11 (25)	33 (75)	1.06	0.48–2.33	0.895			
Purpose of stoma								
• Temporary	40 (26)	117 (75)	1					
• Permanent	3 (30)	7 (70)	1.25	0.31–5.08	0.751			
Timing of surgery								
• Elective	7 (10)	61 (90)	1					
• Emergency	36 (36)	63 (64)	4.65	1.34–8.81	0.002	3.67	2.11–5.77	0.013
Rank of the operating surgeon								
• Junior	40 (30)	93 (70)	1					
• Senior	3 (9)	31 (91)	2.23	1.34–7.25	0.001	4.31	2.32–7.11	0.034
Anatomical location								
•Ileum	8 (47)	10 (53)	1					
• Transverse colon	5 (50)	4 (50)	2.77	1.11–5.34	0.022	4.33	2.15–7.11	0.023
• Sigmoid colon	30 (22)	107 (78)	3.99	2.61–7.22	0.013	0.54	0.11–0.98	0.031
Type of stoma								
• Loop	5 (29)	12 (71)	1					
• Double barreled	29 (25)	86 (75)	1.44	0.45–2.66	0.987			
• End± Hartman procedure	8 (27)	22 (73)	0.56	0.32–4.98	0.056			
• Spectacles	1 (20)	4 (80)	1.65	0. 43–4.11	0.435			

Keys: *OR* Odds ratio, *CI* Confidence interval

### Stoma revision

Stoma- revision was required in 21(17.4%) patients due to stoma-related complications including stomal prolapse (9 patients), stoma retraction (4 patients), intestinal obstruction (2 patients), stoma stenosis (2 patients) and peritonitis (2 patients), necrosis and parastomal hernia one patient each respectively. The length of hospital stay after stoma formation ranged from 5 days to 18 days (mean = 7.4 ± 2.3 days).

### Definitive (curative) surgery

Out of 167 patients, sixty (35.9%) underwent definitive (curative) surgery. Of these, Posterior Sagittal Anorectoplasty (PSARP) which was done in 23 (69.3%) children was the most frequent surgical procedure performed in children. In adults, bowel resection and anastomosis was the leading procedure done in 16 (59.3%) patients. The time interval from stoma formation to definitive (curative) surgery ranged from 1 month to 24 months with a median of 5 months (interquartile, 2 to 7 months) in children and 1 month to 8 months with a median of 4 months (interquartile, 2 to 6 months) in adults (Table 6). A total of 26 (43.3%) post-definitive treatment complications were recorded in sixty patients who underwent definitive (curative) surgery. The most common post definitive treatment complication in children was surgical site infection which occurred in 7 (35%) patients. While in the 6 adults with complications, surgical site infection in 2 (33.3%) patients and enterocutaneous fistula occurred in 2 (33.3%) patients were the leading post definitive treatment complications (Table 7). The majority of definitive surgeries were performed by senior doctors in 46 (76.7%) patients and the remaining 14 (23.3%) definitive (curative) surgeries were performed by junior doctors under direct supervision by senior doctors.

**Table 6:** Distribution of patients according to definitive treatment

Definitive (curative) treatment	Children <18 yrs	Adults >18 yrs	Total N (%)
	N (%)	N (%)	
Bowel perforation repair	4 (12)	8 (30)	12 (20)
Perineal repair	0 (0)	2 (7)	2 (3)
Bowel resection and anastomosis	4 (12)	16 (59)	20 (33)
Pull through	2 (6)	1 (4)	3 (5)
PSARP	23 (69)	0 (0)	23 (38)
Total	33 (55%)	27 (45%)	60 (100)

*PSARP* Posterior Sagittal Anorectoplasty

**Table 7:** Distribution of patients according to post-definitive treatment complications

Post-definitive treatment complications	Children <18 yrs	Adults >18 yrs	Total N (%)
	N (%)	N (%)	
Surgical site infections	7 (35)	2 (33)	9 (35)
Wound dehiscence	3 (15)	1 (17)	4 (15)
Anal stenosis	3 (15)	0 (0)	3 (12)
Entero-cutaneous fistula	1 (5)	2 (33)	3 (12)
Soiling	3 (15)	0 (0)	3 (12)
Peritonitis	2 (10)	0 (0)	2 (8)
Intra-abdominal abscess	1 (5)	1 (17)	2 (8)
Total	20 (77)	6 (23)	26 (100)

### Stoma closure

**Fig. 1:**
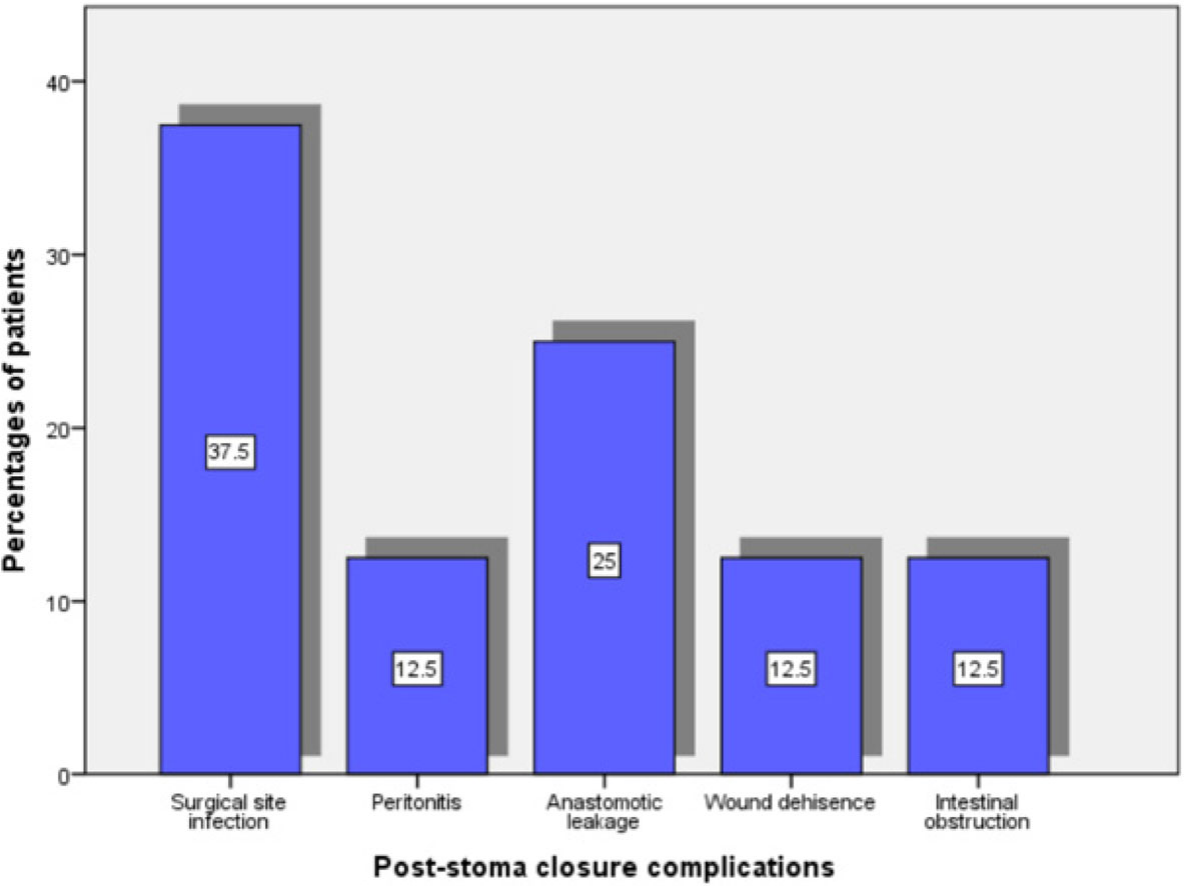
Post-stoma closure complications

A total of 41(19.5%) stomas including 19 ileostomies, 17 sigmoid colostomies, 4 transverse colostomies and 1 jejunostomies were closed at the end of the study period. The time interval from definitive (curative) surgery to stomal closure ranged from 1 month to 6 months with a median of 4 months (interquartile, 2 to 6 months). Of the 41 stomas, 27 (65.9%) were closed extraperitoneally through the stoma site, and the remaining 14 (34.1%) had intraperitoneal closure via a formal laparotomy. The closure was performed by complete resection of the stoma site and end to end anastomosis done in 23 (56.1%) patients and by debridement of the stoma edge and simple closure of the opening in the bowel in 18 (43.9%) patients. The majority of colostomy closure were performed by senior doctors in 34 (82.9%) patients, and the remaining 7 (17.1%) patients had their colostomy closure done by junior doctors under direct supervision of senior doctors, (specialists). A total of 26 (63.4%) different post-stoma closure complications were recorded as shown in Fig. 1. According to multivariate logistic regression analysis, the overall complication rate following stoma closure was found to be significantly higher in stoma closed by junior doctors (OR = 5.23; 95% CI = 2.18–9.86; *p* = 0.001) and in stomas closed intraperitoneally (OR = 6.22, 95% CI = 2.11–8.92; *p* = 0.012).

## Discussion

Since it was first performed more than 200 years ago, intestinal stoma which was created for faecal diversion remains an effective option to treat a variety of gastrointestinal and abdominal conditions [3, 9]. In this study, our patients were found to be significantly younger than the mean of 45 years and 46 years that were reported in similar studies by Nastro et al. [4] and Cheape et al. [10] respectively. This age difference reflects differences in patient populations as former studies were conducted in developed World and majority of participants were adults. Whereas almost more than three quarters of our patients were children, less than 18 years old. Patients reported in studies by Nastro et al. [4] and Cheape et al. [10] were mainly adult population. The male predominance demonstrated in this study was in keeping with previous observations reported in studies performed elsewhere [3, 7, 11]. We could not establish the reason for this age and gender differences.

The preponderance of congenital indications for intestinal stoma formation observed in this study differs from other studies [3, 4] which reported acquired conditions as the most common indication of stoma formation. This preponderance of congenital indications for intestinal stoma formation in the present study, can be explained by the fact that the majority of patients were children less than 18 years old. Similarly, in our study, anorectal malformation was the most frequent indication for stoma formation, contrary to Uddin et al. [11] and Safirullah et al. [12] who reported colorectal cancers as the most common indication for stoma formation. Ahmad et al. [3] reported that the most common indication for stoma formation was the enteric perforation while in the current study, bowel perforation was the second most frequent for intestinal stoma formation. These differences in the indication patterns reflect differences in the disease patterns between the study settings as well as the mean age of the study population.

In agreement with other studies [4, 7] sigmoid colostomy followed by ileostomy were the most frequent intestinal stoma performed in our study. In contrast, Ahmad et al. [3] found that the most common type of ostomy made was ileostomy then sigmoid colostomy and transverse loop colostomy. The same findings seen in a study done by Shah et al. [13] loop ileostomy was the leading stoma done followed by loop colostomy. Ileostomy accounted for 70% stomas in another study by Ghazi et al. [14] followed by a colostomy at 30%. It was reported a study done by Safirullah et al. [12] that loop ileostomy was done in 43% cases while loop colostomy in 17.4% patients. In this study, of the loop stomas, transverse loop colostomies in children accounted for 6.7%, none of the adults had loop stoma, while among the end stomas, sigmoid end colostomies accounted for 71.4 and 37.4% in children and adults respectively. This observation reflects differences in indication patterns for stoma formation between study settings.

Another studies on stoma complications which occurred in the early postoperative period, demonstrated a bit high incidence, which was between 27 and 82% [8–17]. In the present study, the complication rate following intestinal stoma formation was 25.7%, the same finding as it was reported by Husain et al. [18]. The rate of ostomy related complications in this study was significantly high in stomas performed by junior doctors. We could not find the reasons for this finding.

As reported by others [3, 19], many stomas in this current study were done on an emergency basis. Non elective procedures resulted in a high stoma complication rate than elective procedures, and a morbidity was high. These findings are similar to that reported by Stothert et al. [20], who reported more than 50% morbidity and 18% mortality due to emergency surgery resulting in an ostomy formation. An observation suggests, the morbidity and mortality that are caused by emergency surgery may be due to severity of disease and may be due to inadequate preparations of patients.

The ranges of incidence of ostomy prolapse have been documented in the literature varying from 2 to 22% [15, 16, 21]. In this study, stoma prolapse was the commonest complication related to stoma formation accounting for 41.9% of cases, and it was significantly more common in transverse and sigmoid colostomies 66.7 and 55.2% respectively. Many previous studies [7, 22] have reported high rate of ostomy prolapse associated with transverse colostomies. As reported by other reports [7, 22, 23], the proposed reasons for this rate of ostomy prolapse in both transverse and loop colostomies are decrease in diameter of the distal part of distal dilated bowel after stoma formation, mobile transverse and sigmoid large bowels, elevation in a wall and severe malnutrition. Some different operative options have been explained for the purpose of avoiding prolapse, such as skin separation, as well as extraperitoneal pathway of the existing stoma [7, 24, 25]. In our study, only 20.9% of stomas that had severe prolapse causing obstructive symptoms required revision. Other patients with minor to moderate prolapse were managed expectantly until stoma closure.

Surgical site infection and stoma stenosis were reported by Ahmad et al. [3] to have lower rate of 13.4 and 4.0%, while in the current study, the second stoma formation related complication is surgical site infection which accounted for 16.5%, followed by stoma stenosis which accounted for 11.6%. Others [7] reported higher rate of surgical site infection, 32.4%. Many previous studies have reported that the general incidence of stoma retraction varies between 1.4 and 9% [4, 8, 16], and may affect both ileostomies and colostomies [26, 27]. From the findings of this study, the rate of stoma retraction was found to be 7.0% of all stomas, a number that is within the range documented in other studies [4, 8, 16]. Therefore, in order to release tension, detachment of stoma from anterior abdominal wall only is not enough to gain enough length, more exposure is required through big incision to release tension and close ostomy. Other causes of bowel retraction are obesity, bowel wall edema and ischemia in a devascularized segment that was exteriorized. The incidence of other stoma complications such as parastomal hernia, stenosis, bleeding, intestinal obstruction and peritonitis is comparable to other series done elsewhere [6, 7, 22]. Most of these complications were due to poor surgical technique and they needed revision.

Similar to other studies [3, 7], our study found that, stomas performed on emergency basis had significantly higher rate of complications than that performed electively. The high complication rates in emergency stomas may be explained by the fact that, the majority of emergency stomas formation in the present study were performed by junior doctors, and these may have little experiences in forming the stomas. In order to reduce the incidence of complications that follow emergency stoma formation, direct supervision of junior doctors who have not shown true objective proficiency in stoma construction must be enforced as well as strict attention to aseptic technique and meticulous haemostasis.

The closure of stomas has been reported to be associated with significant complications and even mortality and should not be considered as a minor procedure. Complication rates following stoma closure have been reported in the literature to be between 20 and 48% [26, 28, 29]. The post-stoma closure complications rate in our study was 63.4%, a figure which is higher compared to that reported in other studies [6, 7, 30]. We could not find the reason for the high rate of complications following stoma closure in our study.

The optimal timing for the repair of the stoma has been reported to range from four weeks to three months of the initial operation [30]. It has been reported that, if the repair is done earlier than four weeks the risk of anastomotic breakdown is high due to edema, inflammation, and collagenase activity at the site. After three months the stoma becomes firmly adherent to the site due to fibrosis [31]. In our study, the majority of stoma closure were done between 1 month and 6 months. This study illustrates that the optimal time for colostomy closure must be determined on an individual basis.

The technique of colostomy closure has been reported to have an effect on the outcome of patients following stoma closure [30]. In the past, extraperitoneal stoma closure used to be commonly performed with the hope to contain the leak outside the peritoneal cavity. However, recently intraperitoneal closure of stoma is more commonly performed and allows proper identification of the anastomosis under vision [31]. In the present study, approximately two-thirds of stomas were closed extraperitoneally, and this procedure was found to be easy, cost-effective and can be done with minimal complications in our setting.

## Conclusions

The results from this study show that, stoma formation and its closure are commonly performed procedures in our setting and are associated with high complication rate. It is recommended that, emergency patients should be well prepared, the operating surgeon must pay close attention to the fashioning, management and closure of a stoma. Stoma formation and its closure should be done by specialists or junior doctors under direct supervision of specialists to minimize complications related to these operations. Since the transverse colostomy was found to be significantly associated with high complication rates, therefore transverse colostomies should rarely be done and the performance of proximal sigmoid double barreled colostomy with well-fitting stoma bags, proper stoma care, and appropriate timing of stoma closure will improve the outcome of these patients. A close follow up is also highly recommended to be able to detect and manage stoma related complications at an early stage.

## Data Availability

The datasets generated and analyzed during the current study are available from the corresponding author on reasonable request.

## Abbreviations

BMC: Bugando Medical Centre
CUHAS: Catholic University of Health and Allied Sciences
M: R: Male to Female ratio
PSARP: Posterior Sagittal Anorectoplasty
SOPD: Surgical Outpatient Department
SPSS: Statistical Package for Social Sciences
